# The diagnostic accuracy of point-of-care ultrasound parameters for airway assessment in patients undergoing intubation in emergency department—an observational study

**DOI:** 10.1186/s12245-024-00585-6

**Published:** 2024-01-29

**Authors:** Aadya Pillai, Poonam Arora, Ankita Kabi, Udit Chauhan, Reshma Asokan, P. Akhil, Takshak Shankar, D. J. Lalneiruol, Himanshi Baid, Hannah Chawang

**Affiliations:** 1 Department of Emergency Medicine, All India Institute of Medical Sciences, Delhi, India; 2 Department of Emergency Medicine, All India Institute of Medical Sciences, Rishikesh, India; 3 Department of Anaesthesiology, Pain Medicine and Critical Care, AIIMS Gorakhpur, Gorakhpur, India; 4 Department of Diagnostic and Interventional Radiology, All India Institute of Medical Sciences, Rishikesh, India; 5 Department of Emergency Medicine, MGMCRI, Pondicherry, India; 6 Department of Emergency Medicine, Government Medical College Cuddalore, Cuddalore, Tamil Nadu India; 7 Department of Emergency Medicine, Himalayan Institute of Medical Sciences, Dehradun, India

**Keywords:** Difficult intubation, Airway ultrasound, Emergency department

## Abstract

**Background:**

Endotracheal intubation is an essential resuscitative procedure in the emergency setting. Airway assessment parameters such as the Mallampati classification are difficult to perform in an emergency setting. As point-of-care ultrasound (POCUS) assessment of airway parameters does not require patients to perform any mandatory action, ultrasound may become the potential first-line noninvasive airway assessment tool in the emergency department (ED). The use of POCUS in the ED has not been sufficiently studied. Using POCUS in airway assessment for predicting difficult intubation may be the next step in successful airway management.

**Methodology:**

The study was an observational study conducted at the ED of the All India Institute of Medical Sciences (Rishikesh). The treating emergency physician recorded the patient history and systemic examination along with an indication for intubation. The POCUS assessment of airway parameters pre-epiglottis to epiglottic vocal cord ratio (Pre-E/E-VC), tongue thickness, hyomental distance, and distance from skin to the hyoid bone was performed by the study investigator. During laryngoscopy, Cormack-Lehane (CL) grading was assessed. The data was entered and analyzed.

**Results:**

Seventy patients who required intubation in the ED were enrolled in the study. Among the study population, 48.6%, 28.6%, 14.3%, 1.4%, and 7.1% were classified with the following CL grading: 1, 2a, 2b, 3a, and 3b, respectively. At a cutoff of ≥ 1.86, Pre-E/E-VC predicts difficult laryngoscopy (*AUC* 0.835) with a sensitivity of 83% and a specificity of 94%. At a cutoff of ≥ 5.98 cm, tongue thickness predicts difficult laryngoscopy (*AUC* 0.78) with a sensitivity of 83% and a specificity of 88%. At a cutoff of hyomental distance ≤ 6 cm, it predicts difficult laryngoscopy with a sensitivity of 83% and a specificity of 88%. All parameters can act as a promising tool for predicting difficult laryngoscopy, with the single best parameter being Pre-E/E-VC.

**Conclusion:**

Assessment of the airway with POCUS may be helpful to the emergency physician when the clinical airway assessment parameters fail to predict difficult laryngoscopy as most patients requiring intubation are uncooperative. Assessment of the parameters in our study Pre-E/E-VC, tongue thickness, and hyomental distance can act as a promising tool for predicting difficult laryngoscopy in the emergency scenario.

## Introduction

Endotracheal intubation is an essential resuscitative procedure in the emergency setting. Most patients requiring airway management in the emergency department (ED) are critically ill. This presses on the fact that safe and efficient intubations are even more important in the ED. Various mnemonics like LEMON, BONES, and RODS have been studied for quick anatomical assessment of the airway. The practical difficulty of using these parameters in the emergency scenario due to lack of patient cooperation can be resolved using point-of-care ultrasound (POCUS) assessment of the airway.

The incidence of difficult airway in the ED is about 4% according to a study by Wong E. et al. [1]. A retrospective study performed by Levitan et al. in 838 ED patients who underwent intubation to understand the limitations of difficult airway prediction found that Mallampati scoring, neck mobility testing, and measurement of thyromental distance could have been done in only one-third of noncardiac arrest ED intubations and none of the rapid sequence intubation failures [2].

Ultrasound of airway structures is currently used for confirming endotracheal tube placement, identifying the tracheal rings, and locating the site of the surgical airway [3]. As assessment of sonographic parameters does not require patients to perform any mandatory action, ultrasound may become the potential first-line noninvasive airway assessment tool in ED when difficult airway is anticipated.

The difficulty during intubation can be classified according to the CL grading obtained during direct laryngoscopy. As per previous studies, pre-epiglottis to epiglottic vocal cord ratio (Pre-E/E-VC) can predict Cormack-Lehane (CL) grading [4]. A thicker tongue evaluated by ultrasound can also act as an independent predictor for a difficult airway [5]. The other parameters that can be assessed include hyomental distance (HMD) and distance from skin to the hyoid bone (DSHB).

Previous studies on the assessment of the airway with ultrasound were primarily conducted in pre-anesthetic settings, which are a more controlled environment. Its use in the emergency scenario has not been sufficiently studied. Using ultrasound in airway assessment for predicting difficult intubation is the next step in successful airway management.

## Materials and methods

### Study design and settings

The study was an observational study conducted at the ED of the All India Institute of Medical Sciences (Rishikesh) from July 2020 to November 2021. This facility is a tertiary health care center situated in the Indian state of Uttarakhand (Northern India). All patients greater than 18 years of age with an indication of intubation due to inability to maintain airway patency and inability to protect the airway against aspiration, who had failure to ventilate or failure to oxygenate, or with an anticipation of a deteriorating course leading to respiratory failure were included in the study. Patients who require immediate airway management, pregnant women, and those with clinical history suggestive of difficult airway (head and neck burns, neck masses, previous thyroid surgery, and tracheostomy) were excluded from the study.

### Sample size

Calculation was done by Easy ROC software, which is a web-based interface to the R package pROC found at http://www.biosoft.hacettepe.edu.tr/easyROC/.

Keeping the power of 0.8 at a corrected alpha of 0.05, the minimum sample size was calculated as 63. Seventy patients were enrolled in the study.

### Clinical evaluation

The primary treating emergency physician took the patient history, vital signs, systemic examination, and the indication for intubation. Point-of-care ultrasound assessment of airway parameters Pre-E/E-VC, tongue thickness, hyomental distance, and distance from skin to the hyoid bone was performed by study investigator prior to intubation.

### Ultrasound protocol

The study investigator was trained in airway ultrasound for a period of 2 months. Each ultrasound was performed in a systematic manner as planned. The sonography was performed in the red area of emergency, with the patient lying supine and in neutral head position.

**Fig. 1:**
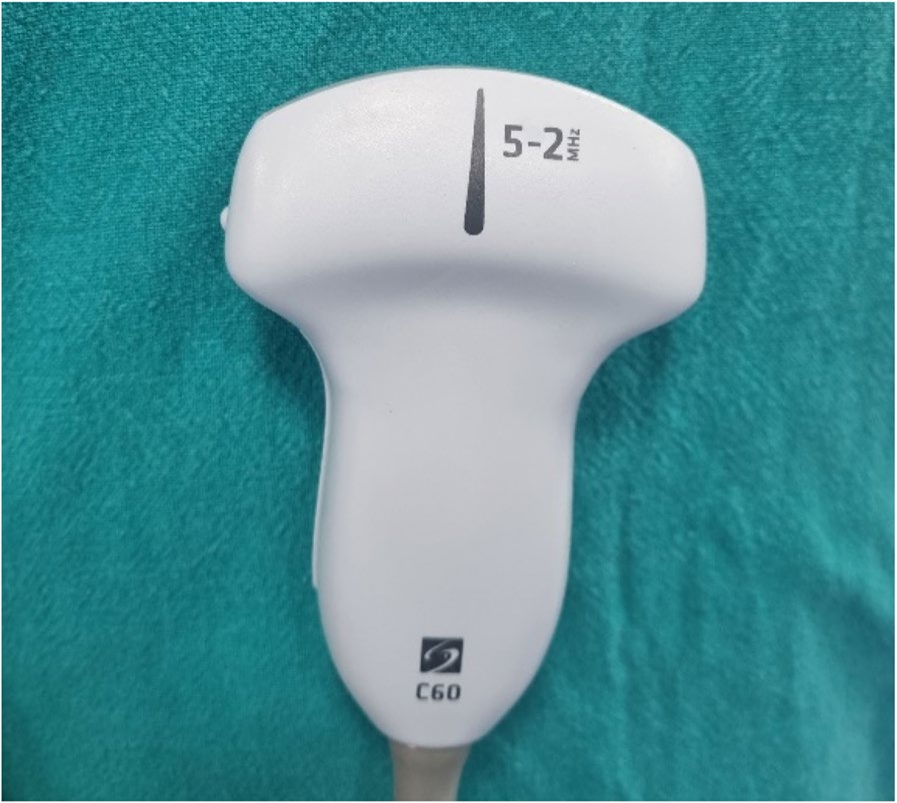
Curvilinear probe

The curvilinear low-frequency (2–5 MHz) transducer (Fig. 1) was used to measure hyomental distance and tongue thickness.

**Fig. 2:**
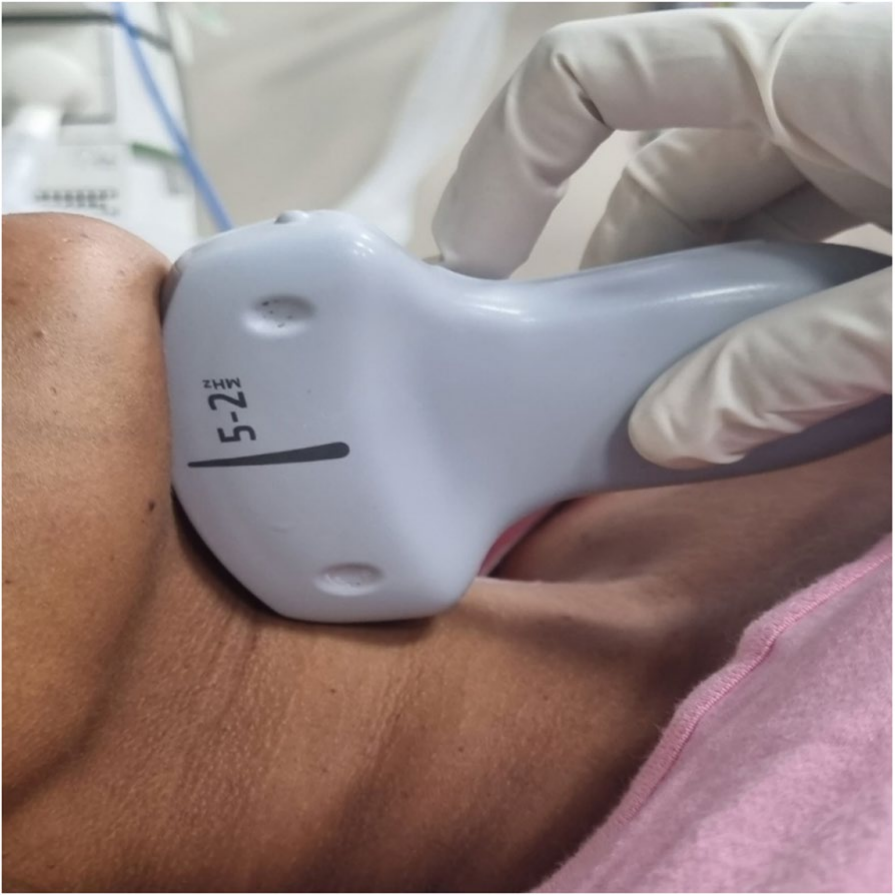
Probe position to measure hyomental distance and tongue thickness

**Fig. 3:**
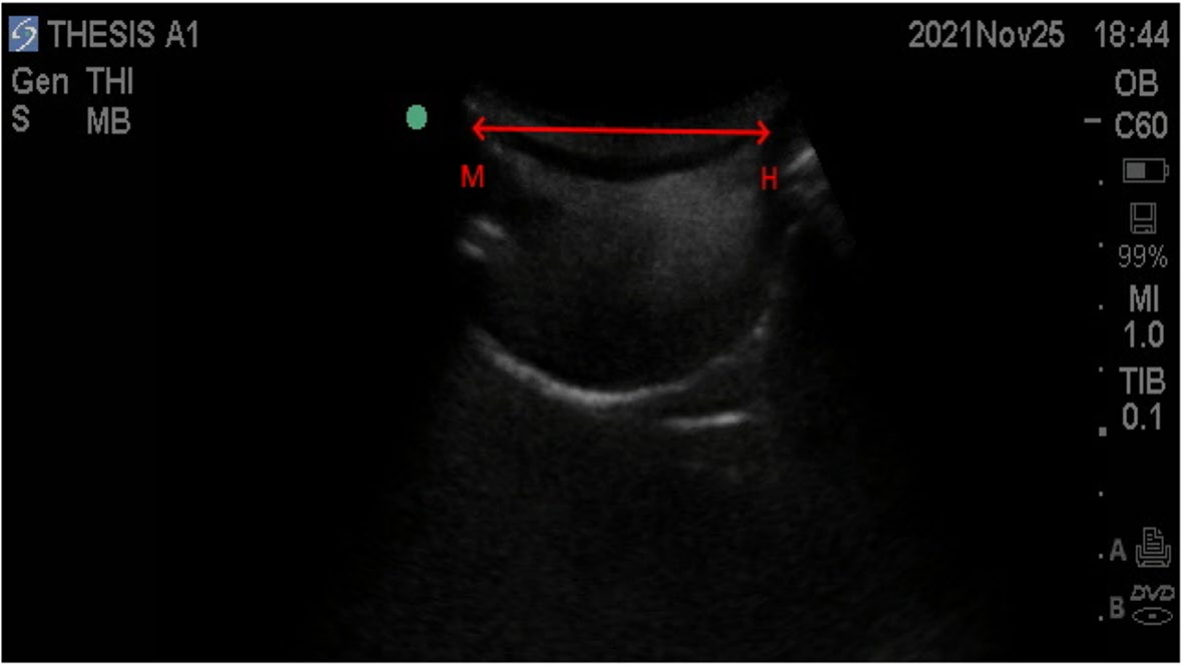
The red arrow represents hyomental distance. M, mentum; H, hyoid bone

**Fig. 4:**
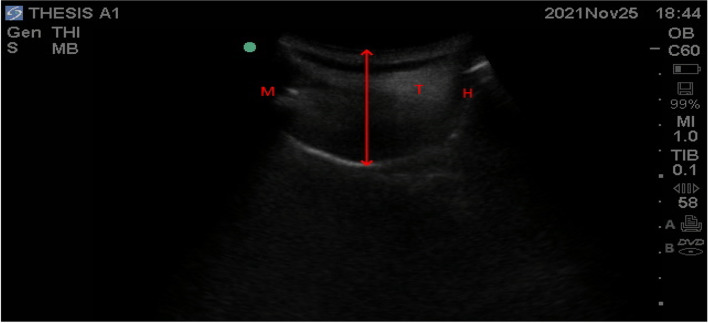
The red arrow represents the tongue thickness. T, tongue; M, mentum; H, hyoid

The curvilinear probe was placed at the lower border of the mandible in the sagittal plane (Fig. 2). Shadow of the hyoid bone, tongue, and mandible was obtained in a single image by maneuvering of the probe. The hyomental distance (Fig. 3) was measured from the upper border of the hyoid bone to the lower border of the mentum in the neutral position followed by measurement of tongue thickness (Fig. 4).

**Fig. 5:**
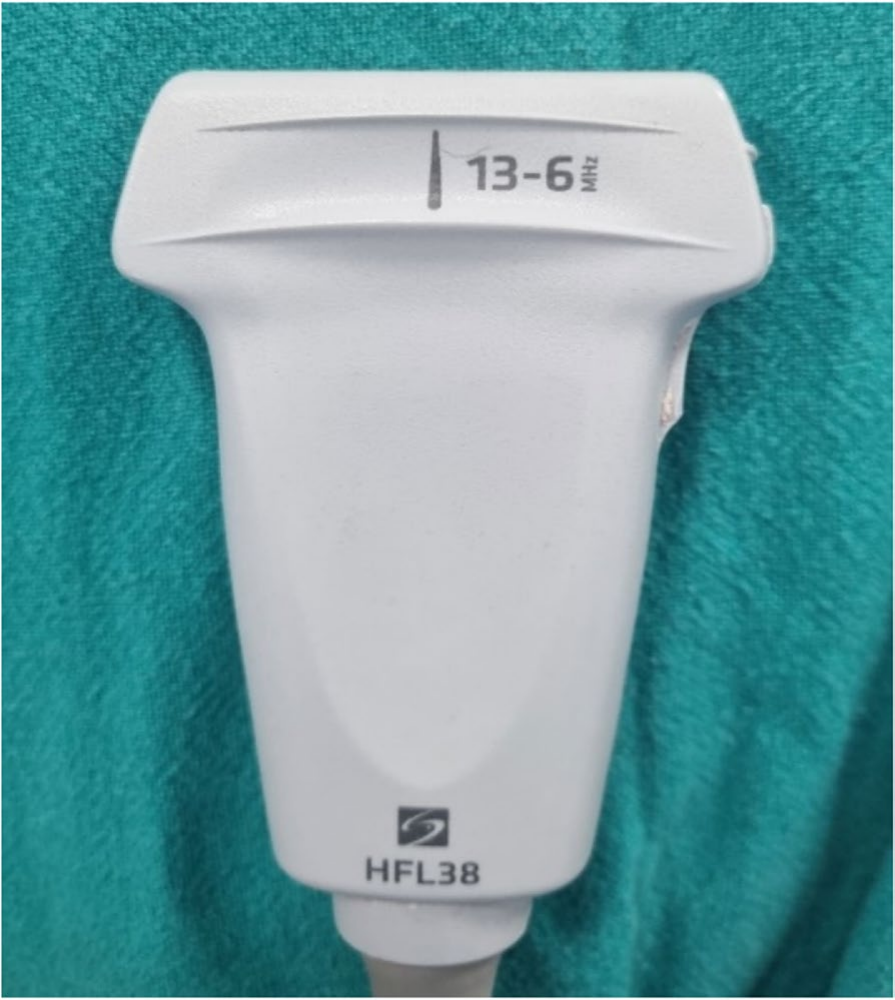
Linear probe

The 6–13 MHz linear probe (Fig. 5) was used to measure the distance from the skin to the hyoid bone and Pre-E/E-VC.

**Fig. 6:**
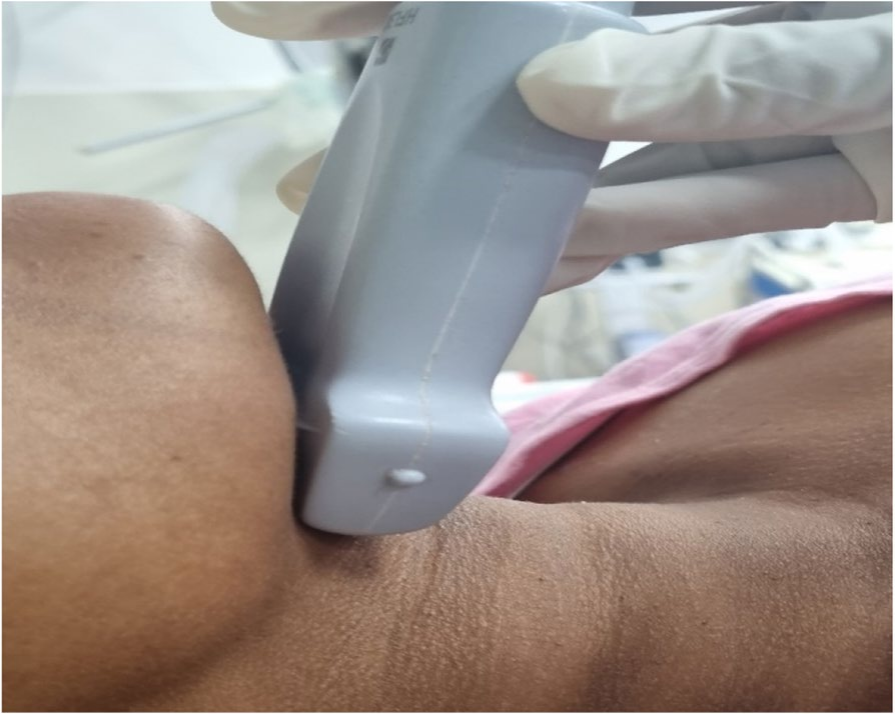
Probe position measuring skin to hyoid bone distance

**Fig. 7:**
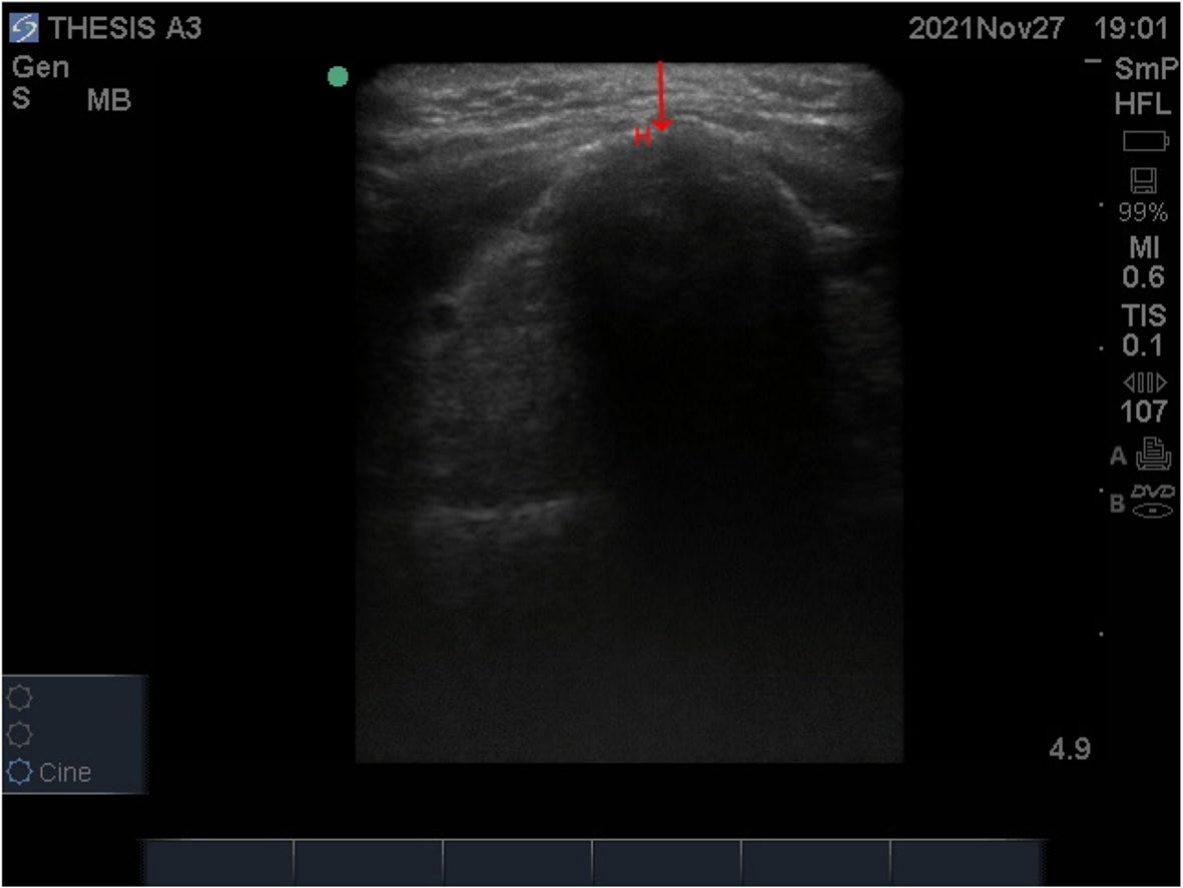
The red arrow represents skin to hyoid bone distance. H, hyoid bone

The linear probe was placed in the transverse plane at the level of the hyoid bone (Fig. 6), and the distance from the skin to the hyoid bone (Fig. 7) was measured.

**Fig. 8:**
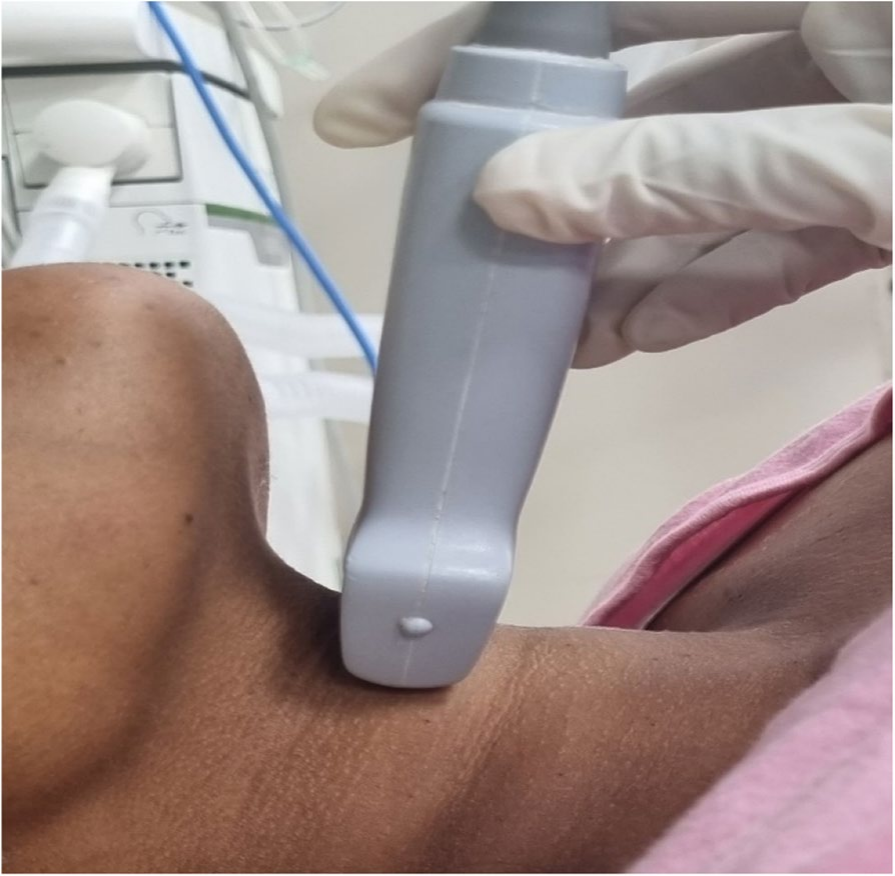
Probe position to measure Pre-E and E-VC distance

**Fig. 9:**
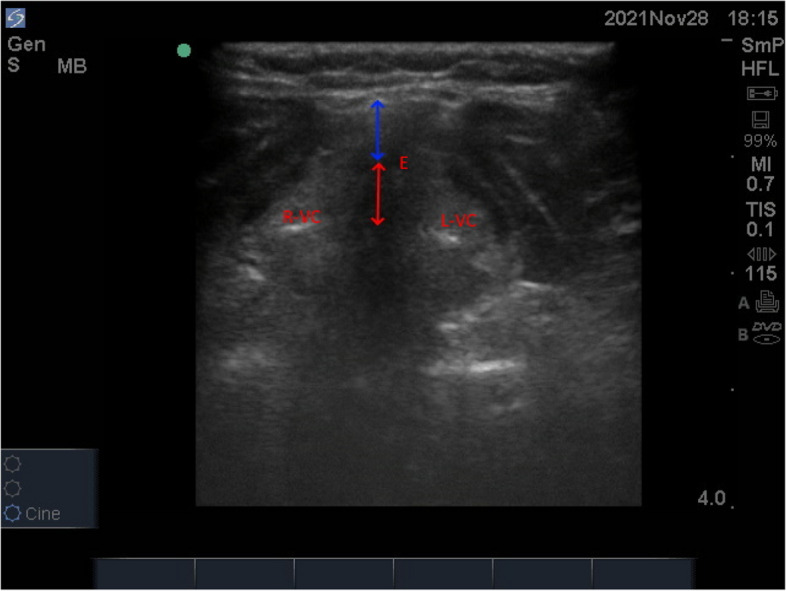
The blue arrow represents pre-epiglottic, and red arrow represents the distance from the epiglottis to the midpoint between vocal cords. R-VC, right vocal cord; L-VC, left vocal cord; E, epiglottis

The linear probe then moved in caudal direction until simultaneous visualization of epiglottis and posterior most part of vocal cords with arytenoids. When this oblique transverse plane view (Fig. 8) that bisects the epiglottis and posterior most part of vocal cords is achieved, the distance from the epiglottis to the midpoint of the distance between the vocal cords (E-VC) and the depth of pre-epiglottic space (Pre-E) was measured (Fig. 9).

### Data collection and analysis

The findings of the ultrasound examination were collected in a standardized form. During laryngoscopy, CL grading was assessed as defined by Cormack and Lehane. CL grade 1 is most of the cords visible, CL grade 2a — posterior cords visible, CL 2b — only arytenoids visible, CL 3a — epiglottis visible and liftable, CL 3b — epiglottis is adherent to the larynx, and CL 4 is no laryngeal structures seen. Difficult laryngoscopy occurs when the view of the laryngeal inlet is poor. Grades 1 and 2 are considered easy, and grades 3 and 4 where the glottis is not visualized are considered difficult laryngoscopy [6].

Ultimately, the ultrasound parameters were correlated with CL grading. The data was entered into an Excel sheet and analyzed with the help of SPSS software version 23.

## Results

Seventy patients who required intubation in the emergency were enrolled in the study. Fifty-five (78.6%) of the participants were males, and 15 (21.4%) of the participants were females. The main baseline characteristics of the study population are shown in Table 1.

**Table 1:** Baseline characteristics of the study population

Characteristics	Values
Age in years, mean (SD)	51.41 (15.54)
Men, number (%)	55 (78.6%)
Women, number (%)	15 (21.4%)
SBP in mmHg, median (minimum-maximum)	120 (70–230)
DBP in mmHg, median (minimum-maximum)	70 (40–120)
Heart rate in beats/min, median (minimum-maximum)	100 (50–190)
Respiratory rate in breath/minute, median (minimum-maximum)	24 (12–56)

Table 1 summarizes the mean age of the study population as 51.41. The study population included 78.6% males and 21.45 females with vitals at value as shown above.

### Indication of intubation

Among the participants, 60% had low GCS as an indication for intubation. A total of 12.9% of the participants had type 1 (hypoxic) respiratory failure, and 17.1% of the participants had type 2 (hypercarbic) respiratory failure. A total of 10.0% of the participants had increased work of breathing as an indication of intubation. The distribution of CL grading is shown in Table 2.

**Table 2:** Distribution of CL grading in the study population

CL grading	Frequency	Percentage
1	34	48.6%
2a	20	28.6%
2b	10	14.3%
3a	1	1.4%
3b	5	7.1%

Table 2 summarizes the distribution of CL grading in the study population with no participants having CL grading 4.

A total of 98.6% of the participants were successfully intubated, whereas 1.4% required a tracheostomy. External laryngeal maneuvers were used in 20% of the participants, and bougie was used in 14.3% of the participants, whereas stylet was used only in 2.9% of the study population.

### Difficult laryngoscopy

**Fig. 10:**
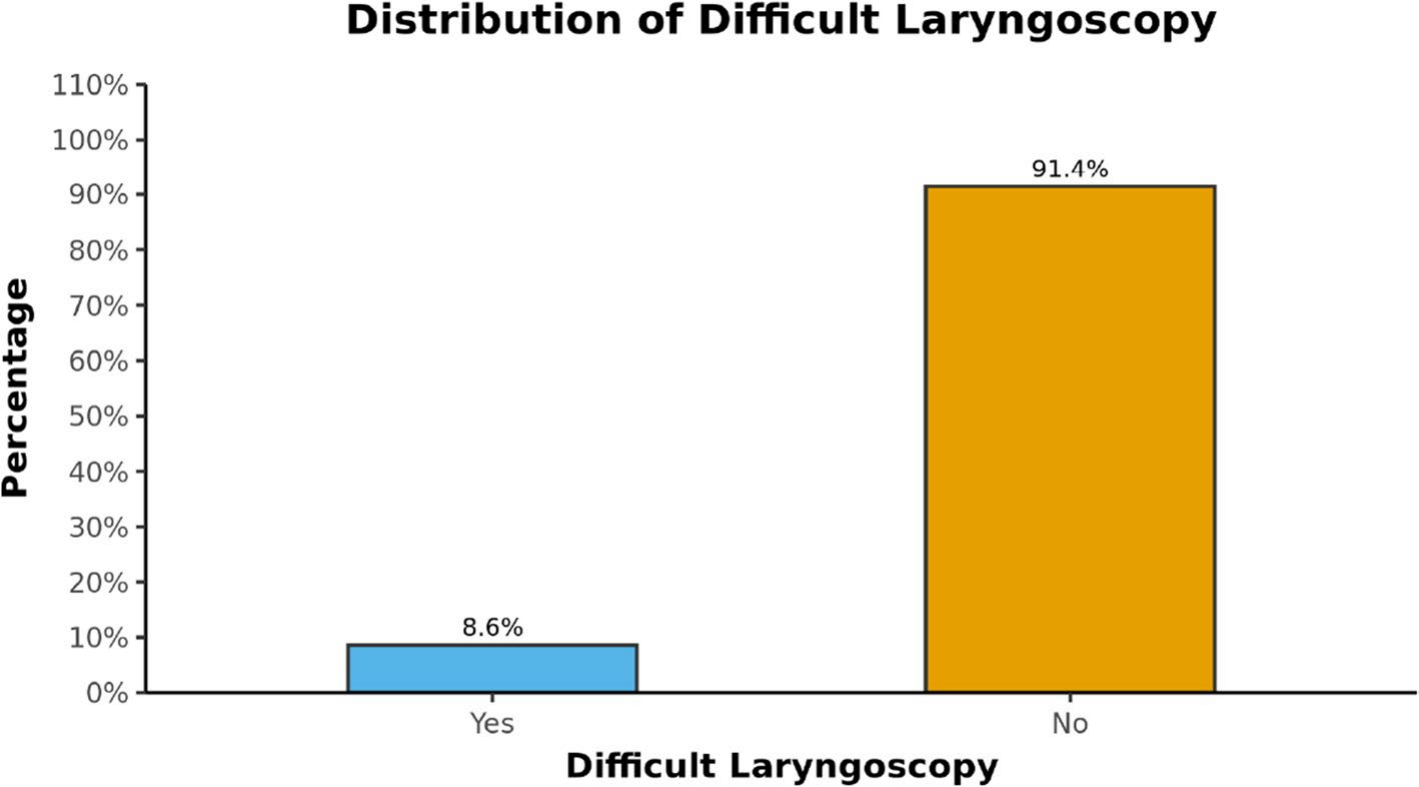
Distribution of difficult laryngoscopy in the study group

Study population was divided into two groups based on CL grading where grades 3 and 4 were considered as difficult laryngoscopy (Fig. 10). Association with ultrasound parameters and their ability to predict difficult laryngoscopy was assessed.

### Association between Pre-E/E-VC and difficult laryngoscopy

The Pre-E/E-VC in the difficult laryngoscopy group ranged from 1.14 to 1.94, whereas the Pre-E/E-VC in the easy laryngoscopy ranged from 0.98 to 1.94. There was a significant difference between the two groups (easy vs difficult laryngoscopy) in terms of Pre-E/E-VC (*W* = 320.500, *p* = 0.007), with the median Pre-E/E-VC being highest in the difficult laryngoscopy group. Strength of association (point-biserial correlation) = 0.36 (medium effect size).

**Fig. 11:**
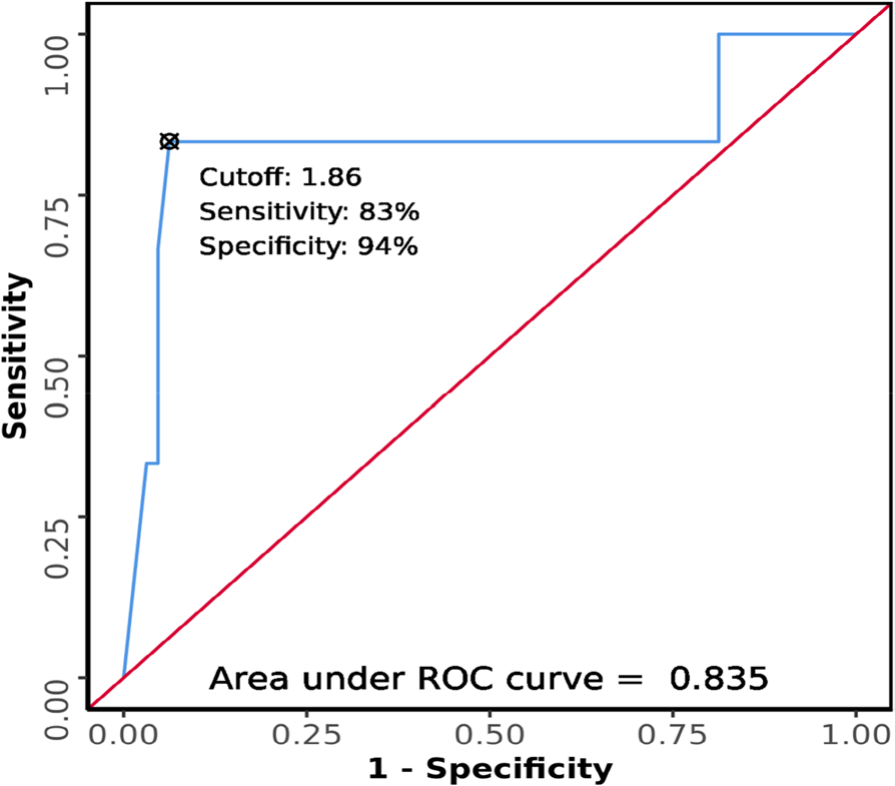
ROC curve analysis of Pre-E/E-VC in predicting difficult laryngoscopy

The area under the ROC curve (AUROC) for Pre-E/E-VC predicting difficult laryngoscopy was 0.835 (95% CI = 0.578–1), thus demonstrating good diagnostic performance (Fig. 11). At a cutoff of ≥ 1.86, Pre-E/E-VC predicts difficult laryngoscopy with a sensitivity of 83% and a specificity of 94%.

### Association between tongue thickness and difficult laryngoscopy

The tongue thickness in the difficult laryngoscopy group ranged from 4.7 to 6.5 cm. The tongue thickness in the easy laryngoscopy ranged from 4.5 to 6.7. There was a significant difference in tongue thickness between the two groups (*W* = 299.500, *p* = 0.025), with the median tongue thickness being highest in the difficult laryngoscopy group. Strength of association (point-biserial correlation) = 0.33 (medium effect size).

**Fig. 12:**
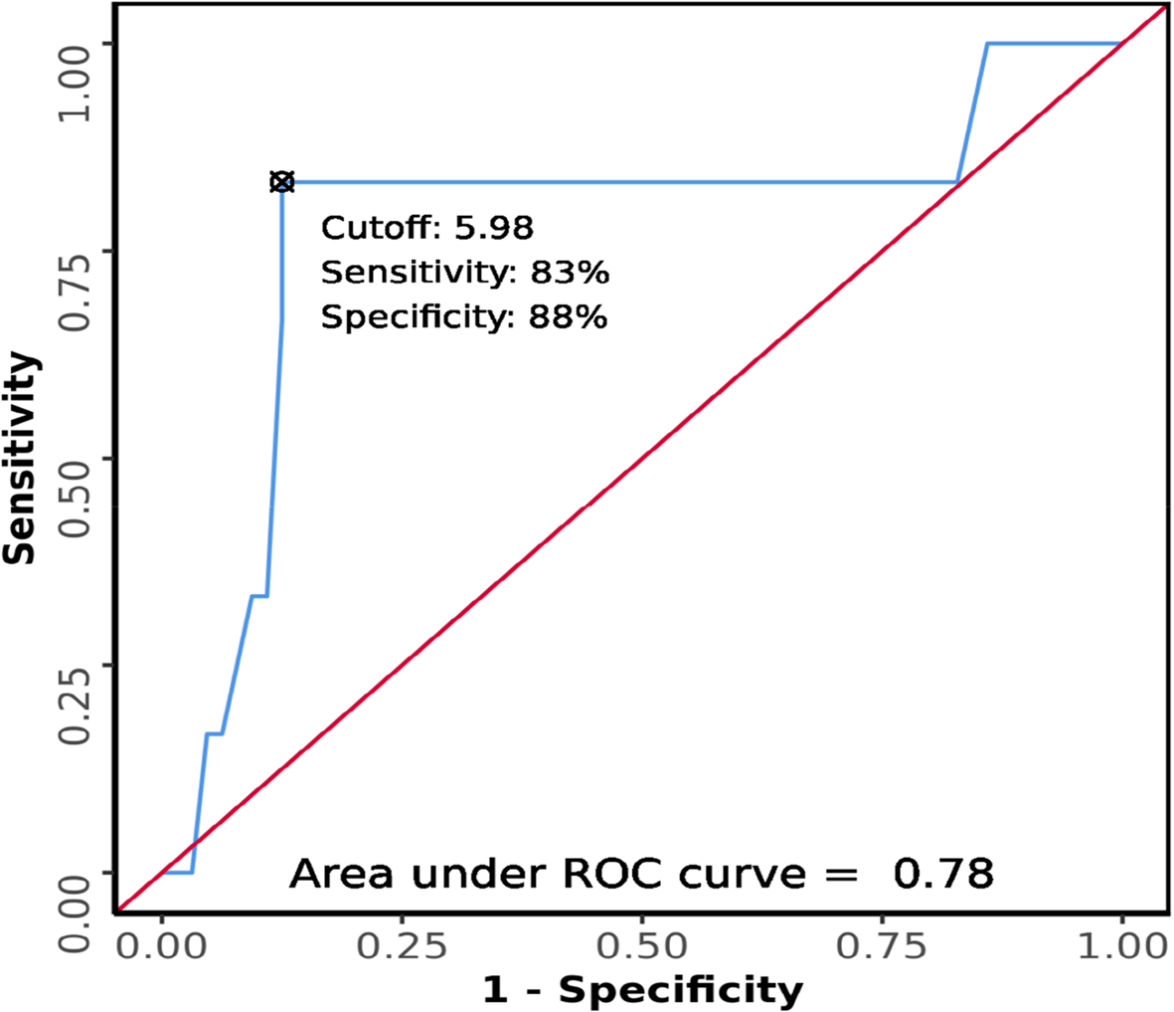
ROC curve analysis of tongue thickness in predicting difficult laryngoscopy

The area under the ROC curve (AUROC) for tongue thickness (Fig. 12) predicting difficult laryngoscopy was 0.78 (95% CI = 0.527–1), thus demonstrating fair diagnostic performance. At a cutoff of ≥ 5.98 cm, tongue thickness predicts difficult laryngoscopy with a sensitivity of 83% and a specificity of 88%.

### Association between HMD and difficult laryngoscopy

The HMD in the difficult laryngoscopy ranged from 5.6 to 7.3 and in the easy laryngoscopy ranged from 4.9 to 7.6. There was a significant difference between the two groups in terms of HMD (*W* = 81.000, *p* = 0.020), with the median HMD being highest in the easy laryngoscopy group. Strength of association (point-biserial correlation) = 0.34 (medium effect size).

**Fig. 13:**
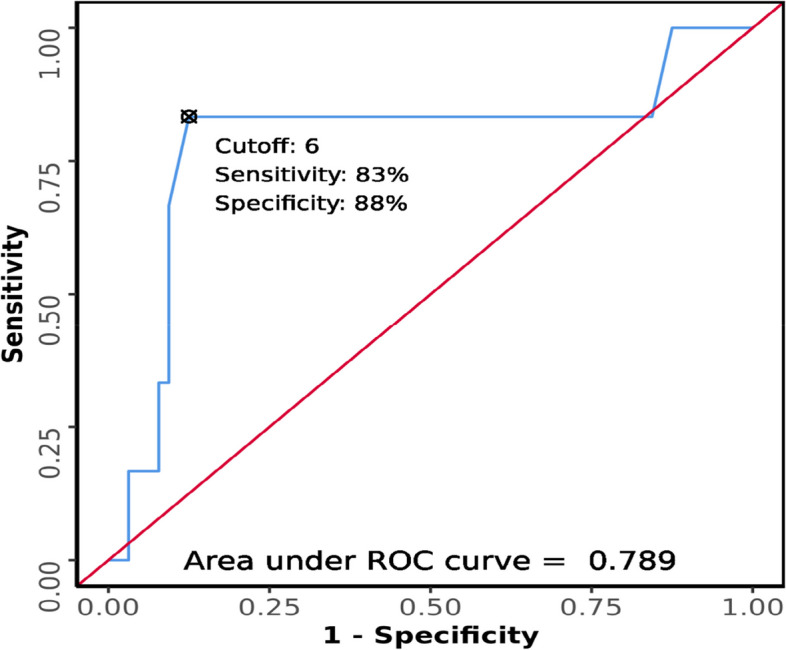
ROC curve analysis of HMD in predicting difficult laryngoscopy

The area under the ROC curve (AUROC) for HMD predicting difficult laryngoscopy (Fig. 13) was 0.789 (95% CI = 0.528–1), thus demonstrating fair diagnostic performance and was statistically significant (*p* = 0.020). At a cutoff of HMD ≤ 6, it predicts difficult laryngoscopy with a sensitivity of 83% and a specificity of 88%.

### Association between DSHB and difficult laryngoscopy

There was no significant difference between difficult and easy laryngoscopy in terms of DSHB (*W* = 270.000, *p* = 0.103).

**Fig. 14:**
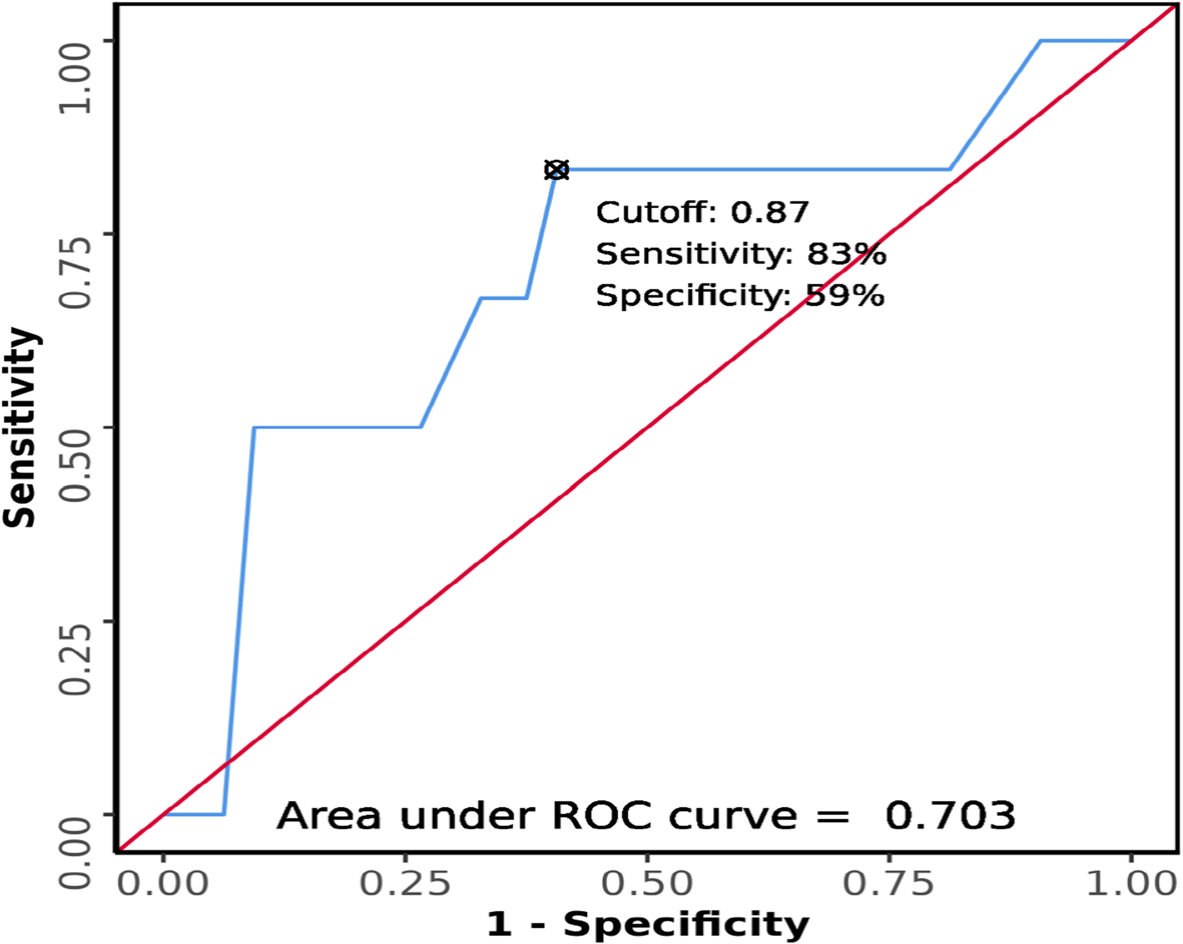
ROC curve analysis of DSHB in predicting difficult laryngoscopy

The area under the ROC curve (AUROC) of DSHB (Fig. 14) for predicting difficult laryngoscopy was 0.703 (95% CI = 0.45–0.956), thus demonstrating fair diagnostic performance. It was not statistically significant (*p* = 0.103). At a cutoff of *DSHB* ≥ 0.87, it predicts difficult laryngoscopy with a sensitivity of 83% and a specificity of 59%. Table 3 compiles the results.

**Table 3:** Cutoff value of POCUS parameters for predicting difficult laryngoscopy

Variables	Difficult laryngoscopy	Sensitivity	Specificity	Diagnostic accuracy
Pre-E/E-VC	≥ 1.86	83.3%	93.8%	92.9%
Tongue thickness	≥ 5.98	83.3%	87.5%	87.1%
Hyomental distance (HMD)	≤ 6	83.3%	87.5%	87.1%
Distance from skin to hyoid bone (DSHB)	≥ 0.87	83.3%	59.4%	61.4%

Table 3 compiles cutoff values for difficult laryngoscopy predicted by Pre-E/E-VC, tongue thickness, hyomental distance, and distance from skin to hyoid bone which were ≥ 1.86, ≥ 5.98, ≤ 6, and ≥ 0.87 cm respectively with maximum specificity for Pre-E/E-VC.

## Discussion

Airway management is an essential skill for an emergency physician. As the patients requiring intubation in the ED are in a life-threatening condition, prompt management of the airway is crucial. The scores generally used in the pre-intubation setting to predict difficulty require patient cooperation, which is not feasible in most patients requiring intubation in an emergency setting.

The majority of the studies on ultrasound assessment of the airway were conducted in a controlled setting like operation theater. Limited data is available in the emergency setting. A feasibility study on ultrasound assessment of the airway in the ED by Hall E. A. et al., comprising 50 patients, measured the tongue base, tongue base to the skin, epiglottic width and thickness, and pre-epiglottic space. These measurements were compared with Mallampati classification and BMI values, of which tongue base and tongue base to skin thickness were found to have linear increase with Mallampati score. All the measurements except epiglottic thickness had a fair to good correlation according to his results [7].

This observational study we conducted evaluated the utilization of POCUS in the assessment of the airway in ED to estimate the diagnostic accuracy of ultrasound parameter Pre-E/E-VC for predicting difficult laryngoscopy. Also assessed was the correlation of Pre-E/E-VC to CL grading. We also studied other sonographic airway parameters, including tongue thickness, hyomental distance, and distance from skin to the hyoid bone. Our study enrolled 70 patients who required intubation and met the inclusion criteria.

Our study population comprised 70 patients with a mean age of 51.41 (15.54) years. The study included 78.6% (55) males and 21.4% [8] females. On laryngoscopy, CL grades 3 and 4 are considered difficult laryngoscopy. In our study, 48.6% had CL grade 1, 43% had CL grade 2, 8.5% had CL grade 3, and no patients had CL grade 4. In a study in preoperative setting by Koundal et al. among 200 patients, 29% had CL grade 1, 58.5% had CL grade 2, 11% patients had CL grade 3, and 1.5% had CL grade 4 [9], whereas lower incidence of difficult laryngoscopy in our study may be attributed to lower sample size (*n* = 70).

The ultrasound parameters Pre-E/E-VC, tongue thickness, hyomental distance, and distance from skin to hyoid bone assessed in our study showed promising results in predicting difficult laryngoscopy. In a study done by Mohammedi S. S. et al., they found only a weak correlation between Pre-E/E-VC and CL grading with a sensitivity of 87% and specificity of 30% [10]. However, our study found a strong positive correlation between Pre-E/E-VC and CL grading (Kendall’s tau = 0.54), with the median being highest in the highest CL grade (*p* < 0.001) which concurred with the results of the other studies.

In our study, ROC for Pre-E/E-VC (*AUC* 0.835 with *p* = 0.007) at cutoff ≥ 1.86 predicts difficult laryngoscopy with a sensitivity of 83% and specificity of 94%. The NPV was 98.4%, and PPV was 55.6%. A study conducted by Rana et al. in a preoperative setting noticed that Pre-E/E-VC had a strong positive correlation with AUC of 0.868 and correlation coefficient of +0.648 (95% CI = 0.798 to 0.938; *p* = 0.00), and the cut-off value of Pre-E/E-VC for predicting difficult laryngoscopy was 1.77 with a sensitivity of 82% and specificity of 80%. The NPV of Pre-E/E-VC was 92.3% and PPV 60.5% [4]. Our study results concurred with this study in terms of sensitivity, NPV, and PPV, whereas specificity was much higher in our study. Higher specificity suggests a Pre-E/E-VC value of less than 1.86 and can reliably predict that patient will not have a difficult laryngoscopy. At the same time, high NPV of 98.4% makes Pre-E/E-VC a reliable parameter to rule out difficult laryngoscopy.

In our study, DSHB and CL grading had a weak correlation (Kendall’s tau = 0.23). At a cut-off value of ≥ 0.87 cm, DSHB predicts difficult laryngoscopy with a sensitivity of 83% and specificity of 59%, but this was not statistically significant (*p* = 0.103). However, Zheng et al., in their study in a general anesthesia setting, found a strong positive correlation between skin to the hyoid bone and CL grading. It showed an AUC of 0.9 with a sensitivity of 85.7% and specificity of 85.1% at cutoff 1.28 cm (*p* < 0.0001) [11]. Longer distances from skin to larynx appear predictive of difficult laryngoscopy, but according to our study, DSHB had a weak correlation with CL grading which may be explained by the geographic difference of the patient population. However, further studies are needed before proceeding with use of DSHB for predicting DL.

The study by Yadav N. K. et al. showed a median tongue thickness of 5.30 cm in easy laryngoscopy and 6.1 cm in difficult laryngoscopy. It predicted difficult laryngoscopy (*AUC* 0.72) with a sensitivity of 71% and specificity of 72% [12]. The median (IQR) value of tongue thickness in our study was 6.35 (6.07–6.39) for difficult laryngoscopy and 5.3 (4.81–5.61) for easy laryngoscopy. In our study, a cutoff tongue thickness ≥ 5.98 could predict difficult laryngoscopy with higher sensitivity (83%) and higher specificity (88%). A moderate positive correlation was observed between tongue thickness and CL grading (Kendall’s tau = 0.46).

Yao W. et al. reported a sensitivity of 75% and specificity of 72% at tongue thickness > 6.1 cm in predicting difficult laryngoscopy (*AUC* 0.69, *p* < 0.001) [5]. Our study results concurred with the results by Xu et al. [13]; according to them, a tongue thickness > 5.86 cm predicts difficult laryngoscopy with a sensitivity = 85% and specificity = 91%. More consistent results with a higher sample size providing level 1 evidence are imperative to mandate its use for routine screening pre-intubation.

Patients with shorter hyomental distances in neutral position were found to be significantly associated with difficult laryngoscopy. Although Petrisor et al. [14] and Wojtczak J. A [15]. did not obtain statistically significant results for this parameter, the overall effect of this measurement was significant (*p* < 0.0001) according to the meta-analysis done by Gomes S. H. et al. [8]. In our study, there was a moderate correlation between HMD and CL grading (Kendall’s tau = 0.32). At cutoff ≤ 6 cm, HMD predicted difficult laryngoscopy with a sensitivity of 83% and specificity of 88% (*AUC* 0.789).

This study is one of the pioneer ones in the use of ultrasound in airway assessment in the emergency department. Despite the fact that it showed promising results, extensive research in the same will provide more evidence. With a bigger sample size and incorporating multiple parameters together can be studied for a better understanding of the airway parameters.

The limitations of the study including the inter-observer variability of ultrasound measurement were not assessed, and the demographic variation of these parameters including race was not taken into consideration.

## Conclusion

Assessment of airway parameters with POCUS is helpful to the emergency physician when the clinical airway assessment parameters fail to predict difficult laryngoscopy as most of the patients are uncooperative. The parameters assessed in our study, Pre-E/E-VC, tongue thickness, and hyomental distance, can act as a promising tool for predicting difficulty in the emergency department, with the single best parameter being Pre-E/E-VC having the highest sensitivity and specificity.

Thus, ultrasound along with its immense use in diagnosis and treatment is a promising tool for effective airway management in the emergency department.

## Data Availability

No datasets were generated or analyzed during the current study.
